# The Bacterial Signature of *Leptospermum scoparium* (Mānuka) Reveals Core and Accessory Communities with Bioactive Properties

**DOI:** 10.1371/journal.pone.0163717

**Published:** 2016-09-27

**Authors:** Wisnu Adi Wicaksono, E. Eirian Jones, Jana Monk, Hayley J. Ridgway

**Affiliations:** 1 Faculty of Agriculture and Life Sciences Lincoln University, Christchurch, New Zealand; 2 Lincoln Research Centre, AgResearch, Christchurch, New Zealand; Universite Paris-Sud, FRANCE

## Abstract

*Leptospermum scoparium* or mānuka is a New Zealand native medicinal plant that produces an essential oil with antimicrobial properties. This is the first study to investigate the structure and bioactivity of endophytic bacteria in mānuka by using a combination of cultivation-independent (DGGE) and dependent approaches. A total of 23 plants were sampled across three sites. Plants were considered either immature (3–8 years) or mature (>20 years). The endophyte community structure and richness was affected by plant tissue and bacterial communities became more stable and uniform as plant maturity increased. A total of 192 culturable bacteria were recovered from leaves, stems and roots. Some bacterial isolates showed *in vitro* biocontrol activity against two fungal pathogens, *Ilyonectria liriodendri* and *Neofusicoccum luteum* and a bacterial pathogen, *Pseudomonas syringae* pv. *actinidiae*. A high proportion of bacterial endophytes could produce siderophores and solubilise phosphate *in vitro*. Gammaproteobacteria was the most variable class, representing the majority of cultivated bacteria with bioactivity.

## Introduction

With the recognition of plants as meta-organisms it is clear that endophytic micro-organisms form a second genome in plants and provide a rich supplementary genetic reservoir to the host, influencing all aspects of plant metabolism. Endophytes are important because of their ability to enhance the plant metabolic repertoire, improve nutrient uptake, protect from pathogens, enhance phytoremediation and increase adaptability of plants toward climate change [1]. International research has demonstrated that endophytic micro-organisms can either directly produce, or modify, metabolites *in planta*. For example, paclitaxel (Taxol®) an anticancer agent produced in *Taxus brevifolian* (yew) can also be produced by the endophytic fungus *Taxomyces adreanae* isolated from the same plant [2]. Several novel antibiotics have also been isolated from endophytic bacteria recovered from medicinal plants [3,4].

*Leptospermum scoparium* J.R.Forst. et G.Forst. var. *scoparium* or mānuka is a New Zealand indigenous shrub. The essential oil and unique honey produced by this plant has drawn considerable attention for commercial purposes due to their potent antimicrobial properties. The mānuka essential oil has antioxidant effects and inhibits the growth of *Bacillus subtilis*, a food spoilage organism and *Trichophyton mentayrophus*, an opportunistic human pathogen that infects skin, nails and hair and causes dermatophytosis [5,6]. Medicinal plants recognised for antimicrobial metabolites may also be a source of endophytes with biocontrol activity or that produce new antimicrobial compounds [7–9]. As a result, study of endophytes recovered from medicinal plants has received some attention by scientists working in this field.

Micro-organisms that commonly associate with a plant species and provide a key role in their physiology may be considered as forming a core microbiome, whereas, others that are region or site specific may be considered as an accessory and only acquired when the plant is grown in a particular environment [10]. Early studies on micro-organisms associated with mānuka revealed that the most common leaf endophyte was a *Phyllosticta* species followed by *Botryosphaeria* spp. and *Alternaria* spp [11]. Another study stated that 450 taxa of fungi (including oomycetes and myxomycetes) may play roles as ectomycorrhizal, mycobionts, endophytes, pathogens, or as saprophytes in mānuka [12]. However, no studies have defined the community structure of endophytic bacteria in mānuka and the bioactivity of members of the endophytic bacteria is unexplored.

As for other medicinal plants, mānuka may harbour endophytic bacteria with bioactive potential. Denaturing gradient gel electrophoresis (DGGE), are often used to analyse bacterial communities without culturing in many different samples and for different purposes. For example, in the rhizosphere of rice grown at different sites, a greater bacterial diversity was observed in the rhizosphere of rice in upland soil compared to lowland soil [13]. In addition, DGGE was used to observe differences in endophyte communities that were related to the ability of plants to resist citrus variegated chlorosis (CVC) caused by *Xylella fastidiosa* [14]. Based on DGGE results, a high abundance of *Curtobacterium flaccumfaciens* was observed in asymptomatic plants and it is likely that this organism plays a role in resistance to CVC. In contrast, although selective for culturable microorganism, cultivation-dependent approaches can provide microbial collections that are useful for bioactivity testing both *in vitro* and *in planta*.

Endophyte communities in NZ native plants recognised for medicinal products have not been explored to date. This research addressed this gap by determining (i) the community structure, and diversity of mānuka endophytic bacteria, (ii) the main factors influencing the composition of mānuka endophytic bacteria, and (iii) whether endophytic bacteria had bioactive potential. In this study, the endophytic bacteria in mānuka were analysed using DGGE in a range of samples from different tissues, sites and plant ages. A combination of cultivation-dependent and independent approaches was applied to reveal new insights into the bacterial endomicrobiome of mānuka.

## Materials and Methods

### Ethics statement

We conducted sampling from three sites i.e. Island Hill Station, West Coast and Travis Wetland. For Island Hill Station and West Coast site, the owner of the land gave permission to conduct the study on this site. For Travis Wetland site, Christchurch City Council gave permission to conduct the study on this site.

### Plant sampling

*Leptospermum scoparium* (mānuka) samples were collected between July and August 2013 in the South Island of New Zealand. The Travis Wetland (-43.484246, 172.690247) is located in Christchurch city and is part of a restored, managed wetland containing plants of the Canterbury chemotype with an average annual rainfall of 500–600 mm. At sampling the roots of mānuka were submerged in 2–5 cm of water. Site 2 was a marginal farm land at Island Hills Station (-42.74402, 172.5617) with an annual rainfall of 600–700 mm. Site 3 was located on the West Coast (-41.93865, 171.4259) and with average annual rainfall of 2400–2600 mm. Samples from six, three and five mature plants (more than 20 years old) and three, two and four immature plants (3–8 years old) were taken from Island Hills Station, Travis Wetland and West Coast sites, respectively. Age was estimated from local knowledge of historic forest fires/ planting dates and by the size/trunk girth of the plants. From each mānuka tree, five different branches and five lateral roots were sampled and stored at 4°C for up to 1 week prior to processing.

### Surface sterilisation of mānuka plant tissue

From each plant a composite sample each of leaf tissue (approximately 20 healthy leaves from five different branches), stem tissue (approximately 0.5–1 cm length of stem from five different branches) and root tissue (approximately 2–3 cm length of five lateral roots) were taken. The samples were surface sterilized prior to DNA extraction using the following procedure: leaves, stems and roots were washed and with stems also being debarked under tap water to remove any adhering dirt then air dried. All plant tissues were soaked in 96% ethanol for 10 s then transferred to 2% sodium hypochlorite solution for 2 min (leaf) or 3 min (stem and root) and rinsed 3 times in sterile water for 1 min. Randomly selected surface sterilised plant tissues (approx. 10% of samples) were pressed onto R2A (Difco, Becton, Dickinson, and Company) and potato dextrose agar (Difco, Becton, Dickinson, and Company) for 30 s as a sterility check.

### Investigation of mānuka endomicrobiome structure using denaturing gradient gel electrophoresis (DGGE)

Propidium monoazide (PMA) is a membrane-impermeant dye that selectively penetrates cells with damaged membranes, which can be considered dead. Once PMA penetrates the cell it intercalates DNA upon exposure to intense visible light and as a result DNA cannot be amplified by PCR [15, 16]. In this study, PMA (Biotium, USA) was used to exclude residual surface DNA from PCR amplification and enrich DNA from the endophytic bacteria fraction.

Surface sterilized plant tissues were treated with PMA before DNA extraction in the following process. Surface sterilized plant tissues were soaked in 500 μL sterile water using transparent 0.7 mL tubes (Axygen, USA) and 1.25 μL 20 mM PMA added. The samples were incubated in the dark for 5 min and then exposed to a 650-W halogen light for 5 min. Total DNA (leaves, stems and roots) was extracted using the PowerPlant™ DNA isolation kit (MoBio Laboratories, Carlsbad, USA) following the manufacturer’s instructions. Stems and roots were crushed and ground to a fine powder with liquid nitrogen prior to DNA extraction.

The V3 hypervariable region of the 16S rRNA gene of bacteria was amplified using primers 341F GC (5’-GC clamp-CCT ACG GGA GGC AGC AG-3’) and 518R (5’- ATT ACC GCG GCT GCT GG-3’) [17]. Group specific bacterial communities i.e Alphaproteobacteria (F203α-L1401 and 341FGC-518R), Betaproteobacteria (Beta359F-Beta682R and 518FGC-Beta682R) and Gammaproteobacteria (Gamma395F-Gamma871R and 518FGC-785R) were amplified using nested PCR as previously described [18–19] with a slight modification for the first PCR for Alphaproteobacteria which was increasing the number of cycles from 20 to 30. PCR was performed using thermal cycler (Applied Biosystem Veriti, Thermofisher Scientific Inc, USA) in a 25 μL volume containing 1x buffer, 0.2 mM deoxynucleotide triphosphates (dNTPs), 1.5 mM MgCl_2_, 0.4 μM of each forward and reverse primers (IDT, Integrated DNA Technologies Inc) and 1 U *Taq* DNA polymerase (Roche, Roche Custom Biotech, Switzerland). A 5 μL aliquot of PCR product was visualised by 1% agarose gel electrophoresis alongside the 1Kb plus DNA Ladder (Invitrogen^TM^, Thermo Fisher Scientific Inc, USA). Gels were stained in ethidium bromide solution (0.5 μg mL^-1^) in 0.5 X TAE (40 mM Tris, 20 mM acetic acid, 1 mM NaEDTA; pH 8.0) for 15 min, destained with water then visualised under ultra-violet light with UVIreader (UVItec Ltd, Cambridge, UK).

DGGE were performed with a Cipher DGGE Electrophoresis System (CBS Scientific). Ten μL of PCR product with 10 μL of loading dye were loaded onto an 8% (w/v) polyacrylamide gel (acrylamide/bis solution, 37.5:1) containing a linear denaturing gradient of 30 to 65% for total bacteria, 40 to 60% for Alphaproteobacteria and Gammaproteobacteria, and 40 to 55% for Betaproteobacteria; 100% denaturant was defined as 7 M urea and 40% (v/v) formamide. The gels were run in 0.5 × TAE buffer for 16 hours at 90 V and 60°C for total bacteria. The time was increased to 18 hours and voltage reduced to 60 V for Alphaproteobacteria, Betaproteobacteria and Gammaproteobacteria. To standardise gels, a single sample (leaf sample from Island Hills Station plant no 1) was added to the first lane of every gel as a marker. The gels were stained by silver staining (0.1% (wt/vol) silver nitrate). Gels were developed with sodium hydroxide and formaldehyde solution (0.01% (v/v)). A fixative solution (40% ethanol, 2% acetic acid in water) was added before the Cairns’ preservation solution (25% ethanol, 10% glycerol in water) and subsequent gel drying.

Analysis of the microbial communities were performed using Phoretix 1D Pro Gel Analysis (Totallab, UK). A matrix based on the presence/absence of bands generated from Phoretix binary data was analysed by Primer version 7 (Primer-E Ltd, Plymouth Marine Laboratory, UK) multivariate software package. Resemblance matrices for community profiles were built by calculating similarities between each pair of samples using Jaccard coefficient [20]. Nonmetric multidimensional scaling (MDS) ordination were generated to interpret multivariate distance between samples and factors. Main and pair-wise PERMANOVA tests followed to test the statistical difference between microbial communities among samples. One band was considered as one bacterial taxa. The number of bands per lane was used as a diversity indicator of the bacterial taxa richness. The bacterial richness was analysed with a general linear model (GLM) to determine the significance of treatment factors and followed by Fisher’s ad-hoc analysis at *P*<0.05 using Minitab 17 (Lead Technologies, Australia).

### Investigation of the mānuka endomicrobiome bioactivity using functionality assays

#### a. Endophytic bacteria isolation from mānuka plant tissues

Surface sterilised tissues were dissected (±1–2 cm) and inoculated onto R2A agar. The agar plates were incubated at 25°C in the dark and observed for colonies growing from the tissue samples every 2–3 days for a period of 4 weeks. The representative bacteria were selected based on differences in colony morphology (shape and colour) [21]. Isolated bacteria were streaked on Nutrient agar (NA) (Oxoid, Thermo Fisher Scientific Inc.) and single colonies were subcultured twice to ensure purity. All recovered bacteria (n = 192) from three different sites were tested using assays for specific bioactivity characteristics. The bacteria were routinely subcultured on NA, at 25°C in the dark for 2 days prior to use in functionality assays. For each assay, duplicate plates or tubes were used for each bacterium.

#### b. Siderophore production

Siderophore production was determined using Chrome Azurol S (CAS) agar plates as previously described [22]. A high throughput screening was done by dividing the plate into four equal sized sections and each section inoculated with a loop of bacteria. The ability of bacteria to produce siderophore was determined by measuring the orange zone around the colony. The orange zone (X) and colony size (Y) was measured in mm in two perpendicular directions using a digital calliper. Subtraction of the average orange zone size from the average colony size (X-Y) produced a final value in mm that was indicative of the siderophore production activity.

#### c. Dual culture assay against fungal and bacterial plant pathogens

The bacteria were tested against the grapevine pathogens *Ilyonectria liriodendri* isolate WP1C and *Neofusicoccum luteum* ICMP 16678 obtained from the Plant Microbiology Group culture collection, Lincoln University. A 6 mm diameter agar disc was taken from a 7-days old culture of *I*. *liriodendri* and a 5-days old of *N*. *luteum* that had been grown on Waksman agar (WA) containing 0.5% peptone (Difco, Becton, Dickinson and Company), 0.5% beef extract (Acumedia, Neogen), 0.5% sodium chloride (Labchem, Thermo Fischer Scientific), 1% glucose (Scharlau, Scharlab S.L.), and 1.5% Difco^TM^ agar (Difco, Becton, Dickinson and Company) and placed in the centre of the agar plate. A loop of each bacterial culture was placed at equidistant points around the pathogen colony (2.5 cm from the centre of WA agar plate). For a control, an agar disc of the pathogen was inoculated onto WA without the bacteria. Antagonistic activity of bacteria was determined by measuring inhibition zone between bacteria and pathogen and by observing pathogen colony morphology after 10 days for *I*. *liriodendri and* 7 days for *N*. *luteum* at 25°C in 12 h light/12 h dark.

A dual culture assay was used to determine inhibition toward the bacterium *Pseudomonas syringae* pv. *actinidiae* (Psa), the causal agent of bacterial canker on kiwifruit. A 100 μL aliquot of 2-day old Psa culture (10^7^–10^8^ CFU mL^-1^) was spread on peptone sucrose agar [23] and a 6 mm filter paper disc dipped in an overnight culture of endophytic bacteria in nutrient broth (NB) (Difco, Becton, Dickinson and Company) was placed on four equidistant points. Antagonistic activity of bacteria was determined after 3 days at 25°C in the dark by measuring the clear zone between the endophyte and Psa.

#### d. Phosphate solubilizing assay using tricalcium phosphate and hydroxyapatite

Phosphate solubilizing ability was determined using agar plates containing insoluble tricalcium phosphate (TCP) [24]. The ability of bacteria to solubilize phosphate was determined by measuring the clear zone around the colony. Inoculation of bacterial isolates and clear zone measurement were done as described for siderophore production after the plates were incubated at 25°C in the dark for 14 days. The phosphate solubilizing agar assay was repeated substituting 0.4% (w/v) TCP with 0.4% (w/v) hydroxyapatite (HA) in the agar.

#### e. Auxin production

Auxin production was determined using Luria Bertani broth containing 5 mM L-tryptophan (Sigma-Aldrich) (LB+Try) [25]. Bacteria were inoculated into 1 mL of LB+Try in a 1.7 mL tube and incubated in the dark at 25°C for 2 days. The growth medium containing each isolate was centrifuged for 5 min at 10, 000 × g to remove bacterial cells. A 500 μL free culture supernatant was mixed with 500 μL Salkowski reagent [26] then incubated at room temperature for 25 min to stabilize the colour change. The ability of bacteria to produce auxin was determined by visually comparing pink colour intensity. The media changed from pale yellow to pink that indicated the presence of auxin due to conversion of L-tryptophan to auxin by the bacteria. To ensure detection was consistent, isolate W9R21, W9R23A, W2MS31, W7R11, bacteria which showed strong, medium, low, and no auxin production respectively, were used as control isolates.

#### f. Identification of culturable bacteria based on 16S rRNA gene

At least two bacteria showing the highest activity from each assay were identified based on the sequence of the 16S rRNA gene. The DNA from each strain was extracted using the rapid REDExtract-N-Amp^™^ Tissue PCR Kit (Sigma-Aldrich; Sigma-Aldrich Co. LLC) according to manufacturer’s instructions. Approximately 1,500 bp of the 16S rRNA gene was amplified using the primers F27 (5’-AGA GTT TGA TCM TGG CTC AG-3’) and R1494 (5’- CTA CGG YTA CCT TGT TAC GAC- 3’) [27]. PCR was performed in a thermal cycler (Applied Bio system Veriti, Life Technologies Ltd, New Zealand) in a total volume of 20 μL containing 10 μL REDExtract-N-Amp PCR Reaction Mix, 4 μL ultrapure water (Life technologies; Thermo Fisher Scientific Inc., USA), 0.4 μM of each forward and reverse primer (IDT, Integrated DNA Technologies Inc., Australia) and 4 μL DNA template. The PCR-amplified 16S rRNA genes were sequenced directly at the Lincoln University Sequencing Facility. The sequences obtained were viewed using Chromas Lite 2.1 (Technelysium Pty Ltd, Australia) and manually trimmed using DNAMAN 4.0 (Lynnon Biosoft, Canada) to remove ambiguous sequence. The sequences were then compared with those of know origin using the Basic Local Alignment Search Tool (BLAST) and the GenBank database (http://www.ncbi.nlm.nih.gov). The sequences were aligned using CLUSTALW and the distance matrices and phylogenetic trees were calculated by neighbour-joining [28] algorithms in MEGA 5 software (Molecular Evolutionary Genetic Analysis) [29]. All sequences were deposited in the GenBank database (http://www.ncbi.nlm.nih.gov) under accession numbers KT968693, KT968694, KT968696, KT968697, KT968699-KT968701 and KU500388-KU500413.

## Results

### Investigation of the mānuka endophytic bacterial community structure using DGGE

#### a. Total bacteria

**Fig 1:**
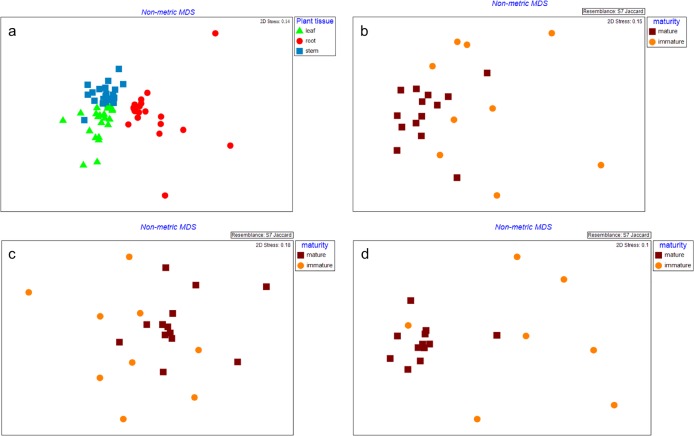
**Nonmetric multidimensional scaling (MDS) plot showing total bacterial communities from (a) different plant tissue and different plant maturities on (b) leaf (c) stem, and (d) root tissue of *Leptospermum scoparium*.** Leaf: green triangle; Stem: blue square; Root: red round; Mature plant: brown square; Immature plant: orange round.

All factors and their interactions influenced total bacterial communities (p≤0.005, Table 1). Leaves, stems, and roots formed discrete clusters independent of geographic origin (Fig 1A). Bacterial communities from mature plants were similar and grouped together, whereas, those from immature plants were diverse, despite common plant tissues (Fig 1B–1D). The number of bands were higher in leaves compared to other tissues (p = 0.001) (Table 2). Richness decreased from leaf>stem>root across all sites. Plant location (p = 0.320) and maturity (p = 0.599) did not influence total bacterial richness (Table 2). Eleven of the DGGE bands were found in ≥90% of the total samples.

**Table 1:** Effect of plant location, plant maturity and plant tissue on the endophytic microbial community similarity using DGGE.

Treatment	^†^ Microbial communities similarity			
	Total bacteria	Alpha proteobacteria	Beta proteobacteria	Gamma proteobacteria
location	0.002**	0.170	0.594	0.001**
maturity	0.001**	0.002**	0.103	0.141
plant tissue	0.001**	0.001**	0.001**	0.001**
location vs maturity	0.001**	0.141	0.290	0.051
location vs plant tissue	0.001**	0.112	0.033*	0.001**
maturity vs plant tissue	0.001**	0.006*	0.001**	0.001**
location vs maturity vs plant tissue	0.001**	0.095	0.084	0.011*

^†^Asterisks denote levels of statistical significance of microbial communities similarity based on PERMANOVA.

*: significantly different (p≤ 0.05)

**: highly significantly different (p ≤0.005)

**Table 2:** Effect of plant location, plant maturity and plant tissue toward microbial richness using DGGE.

Treatment	^¥^Microbial richness			
	Total bacteria	Alpha proteobacteria	Beta proteobacteria	Gamma proteobacteria
location	0.320	0.107	0.784	0.709
maturity	0.599	0.039*	0.202	0.222
plant tissue	0.001**	0.007*	0.076	0.001**
location vs maturity	0.402	0.232	0.896	0.602
location vs plant tissue	0.262	0.313	0.044*	0.092
maturity vs plant tissue	0.492	0.485	0.815	0.013*
location vs maturity vs plant tissue	0.230	0.795	0.196	0.343

^¥^Asterisks denote levels of statistical significance of microbial richness based on GLM.

*: significant different (p ≤0.05)

**: highly significant different (p ≤0.005)

In this study, several DGGE bands from PCR products generated using universal bacteria primers were assigned to chloroplast DNA. These bands were common to all tissues and sites (see S1 Protocol, S1 Table and S1 Fig), therefore, had little influence on community structure and richness of total endophytic bacteria in our samples. This study investigated community structure and richness further using group specific PCR (e.g Gammaproteobacteria) which largely eliminated amplification of chloroplast DNA. Sequencing results from DGGE bands excised from PCR products generated using Gammaproteobacteria specific primers were assigned to the targeted taxa (see S1 Table and S2 Fig).

#### b. Alphaproteobacteria

**Fig 2:**
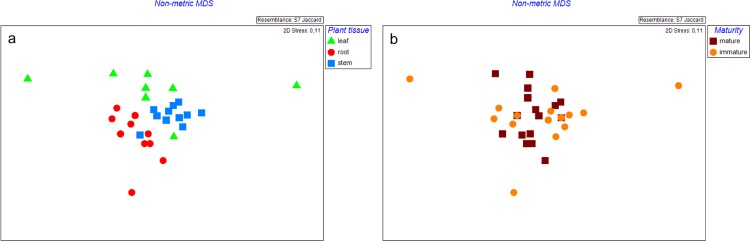
**Nonmetric multidimensional scaling (MDS) plot showing Alphaproteobacterial communities from (a) different plant tissue and (b) different plant maturities of *Leptospermum scoparium*.** Leaf: green triangle; Stem: blue square; Root: red round; Mature plant: brown square; Immature plant: orange round.

Plant tissue and plant maturity influenced Alphaproteobacterial communities (p ≤0.005, Table 1). Stems grouped together, whereas, leaves and roots were more diverse (Fig 2A). Mature plants were more similar to each other compared to immature plants (Fig 2B). Stems and roots had a higher number of bands compared to leaves, with mature plants having a higher number of bands than immature plants (p ≤0.05) (Table 2). Three of the DGGE bands were found in ≥90% of the total samples.

#### c. Betaproteobacteria

**Fig 3:**
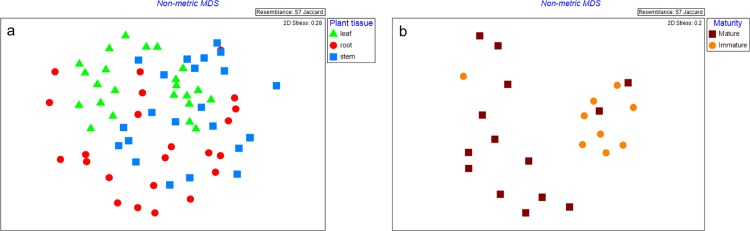
**Nonmetric multidimensional scaling (MDS) plot showing Betaproteobacterial communities from (a) different plant tissue and (b) different plant maturities on leaf tissue of *Leptospermum scoparium*.** Leaf: green triangle; Stem: blue square; Root: red round; Mature plant: brown square; Immature plant: orange round.

Plant tissues and interaction with plant maturity influenced Betaproteobacterial communities (p ≤0.005) (Table 1). Leaves and stems grouped together, whereas, roots were more diverse (Fig 3A). Leaves from mature plant were distinct from leaves from immature plant (Fig 3B). There was an interaction between plant tissue and location where Betaproteobacterial communities in West Coast leaves were different compared to Island Hills Station leaves (p = 0.033). The interaction between plant tissue and location also influenced Betaproteobacterial richness where roots from the West Coast had a lower number of bands compared to other locations (p = 0.044) (Table 2). One of the DGGE bands was found in ≥90% of the total samples.

#### d. Gammaproteobacteria

**Fig 4:**
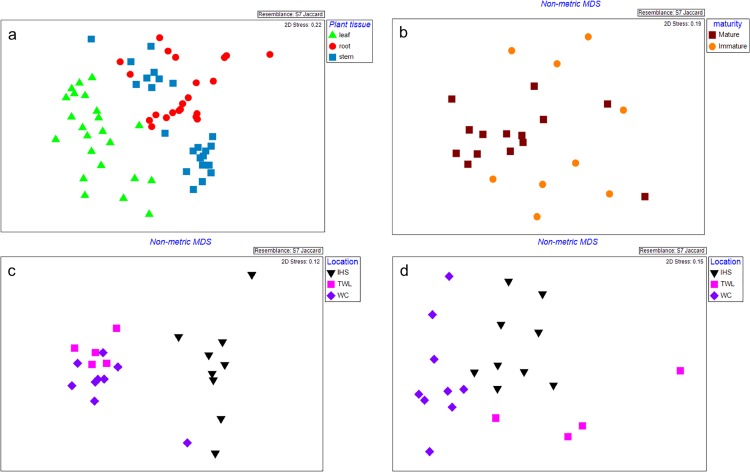
**Nonmetric multidimensional scaling (MDS) plot showing Gammaproteobacterial communities from (a) different plant tissue, (b) different plant maturities on leaf tissue, different plant location on (c) stem and (d) root tissue of *Leptospermum scoparium*.** Leaf: green triangle; Stem: blue square; Root: red round; Mature plant: brown square; Immature plant: orange round; Island Hills Station (IHS): Black inverted triangle; Travis Wetland (TWL): pink square and West Coast (WC): purple diamond.

Plant tissue, plant location, and their interactions with other factors (location vs plant tissue, maturity vs plant tissue, location vs plant tissue vs maturity) influenced Gammaproteobacterial communities (p ≤0.005) (Table 1). Leaves clustered separately from stems and roots samples (Fig 4A). Communities from the leaves of mature plants were distinct from those from immature plant (Fig 4B). Stem communities were similar between the Travis Wetland and West Coast samples and distinct from Island Hills Station, whereas, root samples each formed a cluster based on plant location (Fig 4C and 4D). Leaves had a higher number of bands than other tissues (p = 0.001) (Table 2). Interaction between plant maturity and plant tissue influenced Gammaprotoebacterial richness. Leaves and stems from mature plants had a higher number of bands than roots from mature plant, whereas, leaves from immature plants had a higher number of bands compared to stems and roots from immature plant (p = 0.013) (Table 2). Two of the DGGE bands were found in ≥90% of the total samples.

### Investigation of the mānuka endophytic bacteria bioactivity using functionality assays

A total of 63, 70 and 59 bacteria were recovered from agar plates of plant material taken from Island Hills Station, Travis Wetland, and West Coast sites, respectively. The majority of bacteria (61%; n = 117) was recovered from roots with fewer recovered from stems (30%; n = 58) and leaves (9%; n = 17).

#### a. Bioactivity related to antagonistic activities against plant pathogen

In total, 58% (n = 111) of the bacteria produced siderophores. Plant location influenced the proportion of bacteria producing siderophores. More bacteria from the Travis Wetland and West coast site produced siderophores compared to Island Hills Station (p = 0.001) (Table 3). Based on the plant tissue types, 72% of root bacteria (n = 88), 34% of stem bacteria (n = 20), and 17% of leaf bacteria (n = 3) produced siderophores.

**Table 3:** Proportion of bacteria recovered from *Leptospermum scoparium* that showed siderophore production activity on CAS agar and antagonist effect against *Ilyonectria lirodendri* and *Neofusicoccum luteum*.

Origin site	Siderophore production				Antagonistic against *Ilyonectria liriodendri*				Antagonistic against *Neofusicoccum luteum*			
	-	+	++	+++	-	+	++	+++	-	+	++	+++
Island Hills Station	63.5	9.5	4.8	22.2 ^b^	85.8	6.3	6.3	1.6 ^a^	90.5	6.3	1.6	1.6 ^b^
Travis Wetland	38.6	10.0	14.3	37.1 ^a^	85.7	12.9	1.4	0.0 ^a^	85.7	11.4	2.9	0.0 ^b^
West Coast	23.7	13.6	32.2	30.5 ^a^	76.3	20.3	0.0	3.4 ^a^	61.0	15.3	6.7	17.0 ^a^

The different letter on the last column indicates significantly different activity responses among the sites at p ≤0.05 according Mann-Whitney test. For siderophore production, (-): no clear zone, (+): clear zone less than 5 mm, (++): clear zone 10 mm > x > 5 mm diameter, and (+++): clear zone ≥ 10 mm in diameter; For antagonist effect, (-): no inhibition activity, (+): weak activity–fungal colony showing small indent around bacterial colony but mycelial growth still to the plate edge without visible inhibition zone, (++): moderate activity–fungal growth up to bacterial colony but restricted with inhibition zone < 1mm, (+++): strong activity–fungal growth restricted with inhibition zone > 1mm

In total, 17% (n = 33) of isolates inhibited *I*. *liriodendri* colony growth. There was no difference between sites (p = 0.31) for number of inhibitory isolates or the magnitude of inhibition (Table 3). Only 1.5% of isolates (n = 3) strongly inhibited (group +++) growth of *I*. *liriodendri* and those isolates were all recovered from the roots of plants from Island Hills Station (n = 1) and the West Coast (n = 2). In total, 20% (n = 39) of the isolates inhibited *N*. *luteum* colony growth and 5.2% (n = 10) showed strong inhibition. The West Coast site had more antagonistic isolates compared to other sites (p = 0.001) (Table 3). Island Hills Station site (90.5%; n = 57) had the highest percentage of isolates that did not inhibit pathogen growth. Based on plant tissue type, 25% (n = 31) and 13% (n = 8) of bacteria isolated from root and stem tissue, respectively, inhibited *N*. *luteum* whereas none from the leaf could inhibit this pathogen. The strongest inhibition (group +++) was demonstrated by isolates recovered from root tissue of West Coast site (n = 9) and Island Hills Station (n = 1). Dual culture against Psa showed that only 2.6% (n = 5) of the isolates inhibited Psa. Those isolates were recovered only from Travis Wetland.

#### b. Bioactivity related to plant growth promotion

In total, 66% (n = 128) of bacteria were able to solubilise TCP, whereas 67% (n = 129) of bacteria could solubilise HA across the sites. Roots had a higher proportion of TCP solubilizing bacteria (69%; n = 85) compared to stems (62%; n = 36) and leaves (41%, n = 7). A similar pattern was observed for those bacteria that solubilised HA with 68% (n = 84) recovered from roots, 63% (n = 37) from stems and 47% (n = 8) from leaves. Plant location influenced the proportion of phosphate solubilizing bacteria. The Travis Wetland site had a higher proportion of bacteria able to solubilise TCP compared to other sites (p = 0.001) (Table 4). More bacteria from the Travis Wetland sites could solubilise HA compared to Island Hills Station (p = 0.04) (Table 4). In total, 35% (n = 68) of the bacteria produced auxin. There was no difference between the sites (P = 0.34) (Table 4). Only 1.5% (n = 3) of the isolates showed strong auxin production and all were recovered from the West Coast. More bacteria isolated from stem (36%; n = 21) and root (35%; n = 43) produced auxin, compared to leaf (23%; n = 4).

**Table 4:** Proportion of bacteria isolated from *Leptospermum scoparium* that showed phosphate solubilizing activity on tricalcium phosphate (TCP) and hydroxyapatite and auxin production activity on Luria Bertani broth with 5mM tryptophan.

Origin site	Phosphate solubilizing (TCP)				Phosphate solubilizing (HA)				Auxin production			
	-	+	++	+++	-	+	++	+++	-	+	++	+++
Island Hills Station	41.3	46.0	11.1	1.6 ^b^	34.9	57.2	7.9	0.0 ^b^	71.4	23.8	4.8	0.0^a^
Travis Wetland	18.5	22.9	35.7	22.9 ^a^	30.0	32.9	34.2	2.9 ^a^	58.6	41.4	0.0	0.0 ^a^
West Coast	42.4	23.7	28.8	5.1 ^b^	33.9	45.8	15.2	5.1 ^ab^	64.4	16.9	13.6	5.1 ^a^

The different letter on the last column indicates significantly different activity responses among the sites at p ≤0.05 according Mann-Whitney test. For phosphate solubilizing, (-): no clear zone, (+): clear zone less than 5 mm, (++): clear zone 10 mm > x > 5 mm, and (+++): clear zone ≥ 10 mm in diameter; For auxin production, (-): no activity, (+): low activity (slightly pink colour), (++): moderate activity (moderate pink colour), (+++): high activity (intense pink colour).

#### c. Identification of culturable bacteria based on the 16S rRNA gene

**Fig 5:**
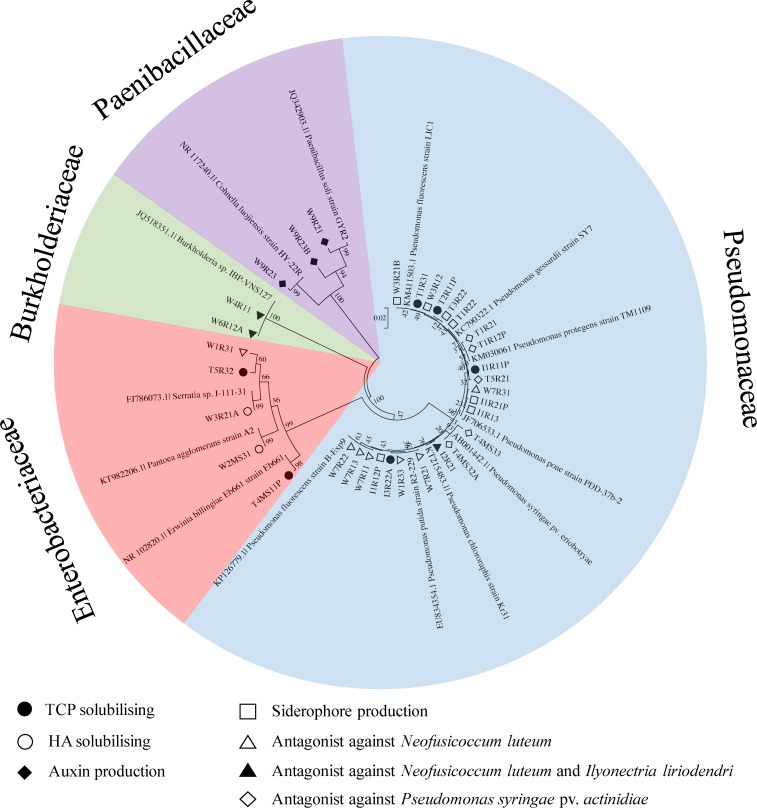
Phylogenetic relationships based on partial 16S rRNA genes of endophytic bacteria from *Leptospermum scoparium* that showed high bioactivities and closely related sequences, based on a distance analysis (neighbour-joining algorithm) 1000 bootstrap replicates performed. Tricalcium phosphate (TCP) solubilizing: filled round; Hydroxyapatite (HA) solubilizing: open round; Auxin production: filled diamond; Siderophore production: Open square; Antagonist against *Neofusicoccum luteum*: open triangle; Antagonist against *Neofusicoccum luteum* and *Ilyonectria liriodendri*: filled triangle; Antagonist against *Pseudomonas syringae* var *actinidiae*: open diamond.

There were four families of bacteria (Pseudomonaceae, Burkholderiaceae, Enterobacteriaceae and Paenibacillaceae) with high bioactivity *in vitro* (Fig 5). Gammaproteobacteria (Pseudomonaceae and Enterobacteriaceae) was the majority of cultivated bacteria with bioactivity (Fig 5). Most bacteria (70%; n = 7) that showed antagonistic activity against fungal pathogens belonged to the Pseudomonaceae, except isolate W6R12A and W4R11 that belonged to the Burkholderiaceae and W1R31 that belonged to Enterobacteriaceae. Those bacteria were recovered from root tissue from the West Coast sites. Only, the Paenibacillaceae showed auxin production activity.

## Discussion

This is the first study to characterise the structure of mānuka endomicrobiome and to demonstrate the bioactivity of culturable bacteria from that community. Mānuka (*Leptospermum scoparium*) is a medicinal plant for which the antimicrobial effects of the unique high triketone oil and honey are well recognized [5, 30–32].

This research confirmed previous work demonstrating that tissue type is a main factor influencing the endophytic microbe diversity and richness in plants [33, 34]. Similar results for bacteria were observed in *L*. *sidoides* [35] and *Stellera chamaejasme* L. (Thymelaeaceae), a medicinal plant that has a wide geographical range [34]. A higher bacterial richness in leaves compared to roots might have been caused by physiological conditions found in each plant organ [34,36]. Roots provide a more stable niche, whereas, leaves provide a limited nutrient supply and are more affected by rapid alteration of environmental conditions which may result in greater heterogeneity of bacterial taxa in leaves compared to roots [34, 37].

Evidence of a core endophytic bacterial community in *L*. *scoparium* was revealed by DGGE. For all three mānuka tissue types there was some overlap in bacterial taxa (DGGE bands that were always found in ≥90% of total samples). Thus, this study has contributed to evidence that different plant organs can share some microbial taxa, defining a core community independent of plant location or maturity. Evidence of a core endomicrobiome was also shown in previous studies on other plants such *as Zea mays* and *Arabidopsis thaliana* [38,39].

This study has demonstrated a common bacterial endomicrobiome in mature plant tissues and shown that these communities became more stable and uniform as the plant matures. Several other studies have shown that microbial diversity and richness change during plant development. For example, the methanogenic archaeal communities in nodal roots of rice [40] and microbial succession in phyllosphere bacteria of lettuce [41]. Changes in the microbial community and richness, especially in leaves, may be linked with essential oil chemistry in mānuka during plant growth. Previous studies on mānuka demonstrated that levels of sesquiterpene and monoterpene differed with plant maturity [42]. In *Lippia sidoides* Cham (Verbenacea) bacterial groups were influenced by the essential oil (thymol and/or carvacrol) composition in the leaves, and the authors suggested that antimicrobial essential oils may place a high selection pressure on the endophyte community [35].

This study demonstrated that plant location not only affected the bacterial community structure and richness but also the proportion of bacteria with the bioactive properties measured. The Gammaproteobacteria were the class most affected by location, represented the majority of the cultivated bacteria and were the most bioactive in functional assays. The sites from which the mānuka was sourced were geographically and environmentally distinct. Plant location comprises a complex range of environmental and abiotic factors that can affect microbial community structure in the rhizosphere and within plants [33, 43, 44]. From DGGE data and comparison of bioactive properties, the bacterial community at Island Hill Station differed compared to other sites. Island Hill Station is considered marginal land with low fertility soils and thus, it might be expected to have different proportion of bacteria with specific properties, such as phosphate solubilizing bacteria, or different Gammaproteobacterial community when compared to more fertile sites such as the Travis Wetland or West Coast site.

In this study, DGGE was used to explore factors affecting the structure of endophytic bacterial communities. Although a well-recognized and widely used technique several limitations are attributed to DGGE when assessing community diversity including the inability to detect minor components of the microbial community [19], co-migration of DNA molecules with different sequences and the potential to produce multiple bands from a single bacterial species [17, 45, 46]. These facets may result in an over or under estimate of the community diversity. This could be resolved using high throughput sequencing to identify species with greater accuracy [47, 48]. However, despite its limitations, several studies have demonstrated a congruent pattern of bacterial composition and diversity between DGGE and high throughput sequencing [48–52]. Therefore, DGGE could provide a robust and quick approach to overview compositional variation in bacterial communities [49].

This study is the first to isolate and test the bioactivity potential of cultured endophytic bacteria from a native medicinal plant in New Zealand. In this study, a high number of bacteria with antagonistic activities against fungal and bacterial pathogen were recovered. The frequency was similar in ginseng where 24% (n = 8) and 18% (n = 6) of endophytic bacteria inhibited growth of *Pythium ultimum* and *Phytophthora capsici*, respectively [53]. In this study, strongest activity was demonstrated by *Pseudomonas* and *Burkholderia* genera which are known producers of antibiotics such as 2,4-diacetylphloroglucinol, pyrrolnitrin, pyoluteorin, and phenazine [54–56]. Disease control using endophytic bacteria is a promising approach [57–59] and the mānuka endomicrobiome may be a new source of candidate biocontrol agents. Culturable bacteria also demonstrated plant growth promotion traits, such as, ability to solubilise phosphate. That result was consistent with other studies on medicinal plants, such as, those on snow lotus (*Saussurea involucrata*) and *Glycyrrhiza* spp. [60, 61].

In summary, this study has demonstrated for the first time that a tissue specific core endomicrobiome forms in mature specimens of mānuka. The presence of a core microbiome suggests it is likely to be important to the physiology of the plant. Gammaproteobacterial communities were influenced by location suggesting this group might play important roles for site specific nutrient uptake or plant protection The rich and unexplored endomicrobiome of mānuka may represent a novel source of antimicrobial agents.

## Supporting Information

S1 Protocol Sequencing of DGGE bands and sequence analysis. (DOCX)

S1 Table Sequence analysis of bands retrieved from endophytic DGGE patterns. The band were sliced from universal bacteria DGGE and Gammaproteobacteria DGGE.
(DOCX)

S1 Fig Representative DGGEs of total bacteria from different tissue types. I: Island Hill Station; T: Travis Wetland; W: West Coast. Arrow indicating selected band that excised and sequenced.
(TIFF)

S2 Fig Representative DGGEs of Gammaproteobacteria from leaves tissue with different maturities. I: Island Hill Station; T: Travis Wetland; W: West Coast. Arrow indicating selected band that excised and sequenced.
(TIFF)
