# Supplementary material for: The Bacterial Signature of Leptospermum scoparium (Mānuka) Reveals Core and Accessory Communities with Bioactive Properties
Source: PLoS One. 2016 Sep 27;11(9):e0163717. doi: 10.1371/journal.pone.0163717 (PMC5038978; doi:10.1371/journal.pone.0163717)
Supplement: S1 Protocol — (DOCX) [file pone.0163717.s001.docx]

**S1 Protocol. Sequencing of DGGE bands and sequence analysis**

A number bands (n=12, see Fig. S1 and S4) in the different DGGEs were sequenced for identification purposes. The DGGE bands from PCR products generated using universal bacterial and Gammaproteobacterial primers (to confirm specificity of the group specific bacteria primers) were excised from the gel, macerated in 50 µL sterile water with sterile pipette tips, incubated at 100°C for 5 min and submitted to a further PCR amplification according to the protocol previously described for PCR-DGGE in the present article. Amplicons were sequenced directly at the Lincoln University Sequencing Facility. The sequences were then compared with those of know origin using the Basic Local Alignment Search Tool (BLAST) and the GenBank database (http://www.ncbi.nlm.nih.gov).
