# Supplementary material for: The Bacterial Signature of Leptospermum scoparium (Mānuka) Reveals Core and Accessory Communities with Bioactive Properties
Source: PLoS One. 2016 Sep 27;11(9):e0163717. doi: 10.1371/journal.pone.0163717 (PMC5038978; doi:10.1371/journal.pone.0163717)
Supplement: S1 Table — The band were sliced from universal bacteria DGGE and Gammaproteobacteria DGGE. (DOCX) [file pone.0163717.s002.docx]

| DGGE band | Group of bacteria | | Closest match | | Accession # | | Similarity | |
| --- | --- | --- | --- | --- | --- | --- | --- | --- |
| L1 | Universal bacteria | *Panax stipuleanatu*s chloroplast | | KX247147.1 | | 100% | |  |
| L2 | Universal bacteria | | *Panax stipuleanatus* chloroplast | | KX247147.1 | | 100% | |
| S1 | Universal bacteria | | *Panax stipuleanatus* chloroplast | | KX247147.1 | | 100% | |
| S2 | Universal bacteria | | *Panax stipuleanatus* chloroplast | | KX247147.1 | | 100% | |
| R1 | Universal bacteria | | *Panax stipuleanatus* chloroplast | | KX247147.1 | | 99% | |
| R2 | Universal bacteria | | *Panax stipuleanatus* chloroplast | | KX247147.1 | | 100% | |
| G1 | Gammaproteobacteria | | Uncultured bacterium | | AB696180.1 | | 97% | |
| G2 | Gammaproteobacteria | | Uncultured *Acinetobacter* sp. | | KC009939.1 | | 100% | |
| G3 | Gammaproteobacteria | | *Pseudomonas migulae* | | AY605698.1 | | 99% | |
| G4 | Gammaproteobacteria | | *Pseudomonas antarctica* | | KX417149.1 | | 98% | |
| G5 | Gammaproteobacteria | | Uncultured Gamma proteobacterium | | EF665429.1 | | 100% | |
| G6 | Gammaproteobacteria | | Uncultured Gamma proteobacterium | | FM209125.1 | | 97% | |

**S1 Table. Sequence analysis of bands retrieved from endophytic DGGE patterns.** The band were sliced from universal bacteria DGGE and Gammaproteobacteria DGGE
