# Supplementary figures and images for: The Bacterial Signature of Leptospermum scoparium (Mānuka) Reveals Core and Accessory Communities with Bioactive Properties
Source: PLoS One. 2016 Sep 27;11(9):e0163717. doi: 10.1371/journal.pone.0163717 (PMC5038978; doi:10.1371/journal.pone.0163717)

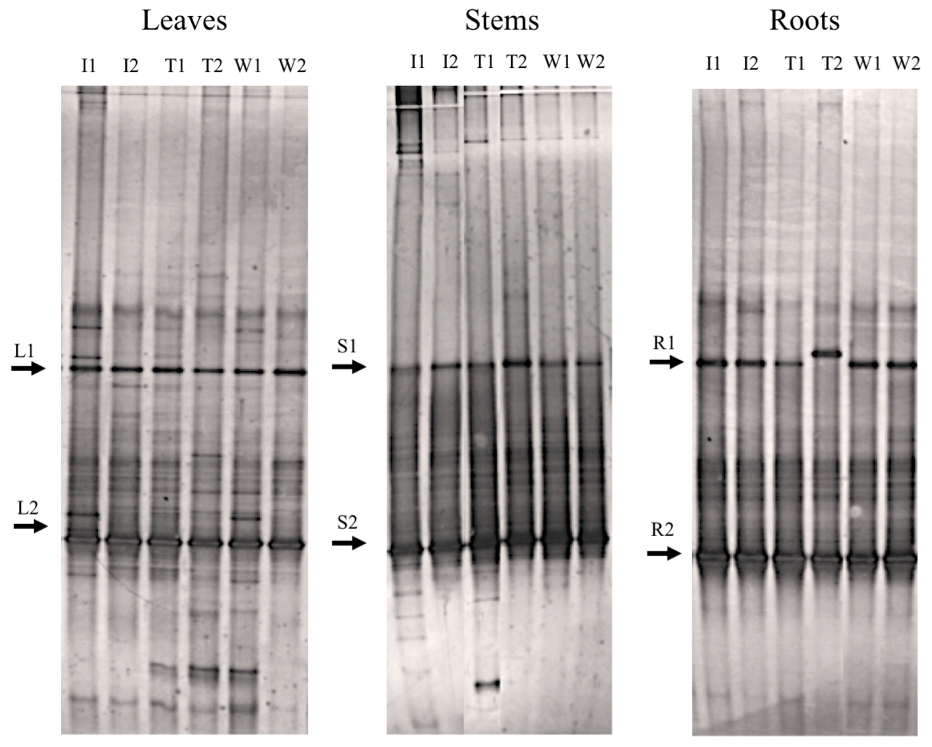

Supplement: S1 Fig — I: Island Hill Station; T: Travis Wetland; W: West Coast. Arrow indicating selected band that excised and sequenced. (TIFF) [file pone.0163717.s003.tiff]

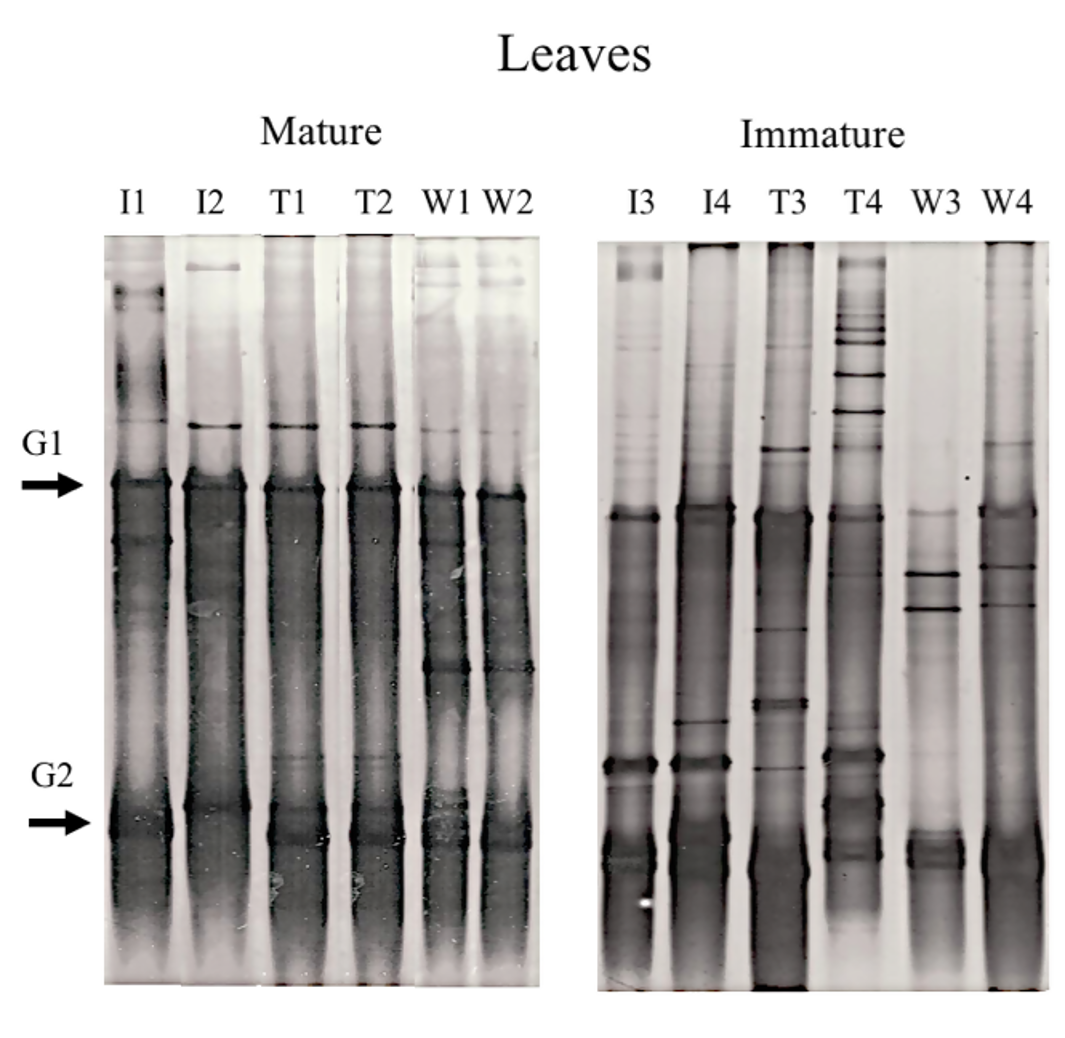

Supplement: S2 Fig — I: Island Hill Station; T: Travis Wetland; W: West Coast. Arrow indicating selected band that excised and sequenced. (TIFF) [file pone.0163717.s004.tiff]
